# Analysis of Factors Associated With Recurrence of Early-Stage Endometrial Carcinoma and Atypical Endometrial Hyperplasia in Infertile Women After *In Vitro* Fertilization Treatment

**DOI:** 10.3389/fonc.2022.892995

**Published:** 2022-06-27

**Authors:** Yaxing Guo, Xuan Zong, Hongzhen Li, Jie Qiao

**Affiliations:** ^1^ Center for Reproductive Medicine, Department of Obstetrics and Gynecology, Peking University Third Hospital, Beijing, China; ^2^ National Clinical Research Center for Obstetrics and Gynecology, Peking University Third Hospital, Beijing, China; ^3^ Key Laboratory of Assisted Reproduction (Peking University), Ministry of Education, Beijing, China; ^4^ Beijing Key Laboratory of Reproductive Endocrinology and Assisted Reproductive Technology, Peking University Third Hospital, Beijing, China

**Keywords:** endometrial carcinoma, atypical endometrial hyperplasia, assisted reproductive technology, recurrence, *in vitro* fertilization

## Abstract

**Purpose:**

To explore the relationship between different artificial reproductive treatment (ART) strategies and tumor outcomes, by analyzing clinical data of patients with endometrial carcinoma (EC) and atypical endometrial hyperplasia (AEH).

**Methods:**

This retrospective study was performed in a tertiary hospital. Patients (n=131) with EC or AEH, who underwent *in vitro* fertilization (IVF)/intracytoplasmic sperm injection (ICSI) treatment between June 2010 and June 2021, were divided into a recurrence group and a non-recurrence group. Clinical characteristics and tumor outcomes were assessed.

**Results:**

131 patients were followed up for 4-132 months; 33 patients had recurrence, the recurrence rate was 25.2%, 3-year recurrence-free survival (RFS) rate was 83.2 ± 3.4%, and the 5-year RFS rate was 72.9 ± 4.4%. Factors including the frequency of controlled ovarian stimulation (COS) and the total days of ovarian stimulation had no significant effect on the recurrence of tumor lesions (p=0.368 and 0.969, respectively). Histology type (HR: 4.94, 95%CI: 2.41-10.15, *p <*0.001) and successful/un successful live birth (HR: 0.30, 95%CI: 0.14-0.65, *p*=0.003) were independent factors of recurrence. Twenty-two of the 82 patients who received a single COS had recurrence. Different COS protocols, the total dose of gonadotropin (Gn), and the serum E_2_ level on the trigger day had no significant effect on recurrence (*p*=0.326, 0.889 and 0.468, respectively).

**Conclusions:**

The degree at which an endometrial lesion progresses into carcinoma is a key factor affecting the recurrence of EC/AEH in patients after IVF/ICSI treatment, and successful live birth is a protective factor for the recurrence of endometrial lesions. Different COS protocols and COS frequencies, as well as the dosage and duration of Gn used during IVF did not affect the recurrence of endometrial lesions.

## Introduction

Endometrial cancer (EC) is one of the most common gynecological malignancies worldwide, with more than 410,000 new cases in 2020 (1). Atypical endometrial hyperplasia (AEH) is a precancerous lesion of endometrial carcinoma whose malignant transformation rate is 29%-52% (2). Although EC is often seen in postmenopausal women, approximately 5% of patients are diagnosed before age of 40 years, which includes 70% of childless women (3). The standard management for EC/AEH is hysterectomy and bilateral salpingo-oophorectomy which is not suitable for young patients with fertility desire (4). The effectiveness of conservative treatment in young patients with early-stage endometrial carcinoma (EEC) and AEH has been confirmed with a high complete remission (CR) rate (75-96.5%). However, the rate of recurrence is as high as 26.0-40.6%, and the median recurrence time was 12-28 months (5–7). Patients who underwent standard management for EEC/AEH had better prognosis, with 5- and 10-year survival rates of 99.2% and 98%, respectively (5). The challenge for EEC/AEH patients is how to get a livebirth as early as possible and then to receive the standard management. However, repeated intrauterine operations will lead to increased incidence of thin endometrium and intrauterine adhesions. Ovarian reserve and patients’ fertility decreased with the growth of age. In addition, most patients with EC/AEH may have combined factors, such diabetes or obesity that may lead to infertility. In order to successfully achieve a livebirth as soon as possible before the recurrence of the disease, the use of artificial reproductive treatment (ART) has become the first choice for most doctors and patients. Indeed, many reports have confirmed the effectiveness of EC/AEH patients using ART for pregnancy, and the live birth rate of ART was 6.9 times than that of natural pregnancy (8). Furthermore, Zhou (9) observed that the clinical pregnancy rate of ART was significantly higher than that of natural pregnancy (72.7% vs 10.0%, p=0.04). Thus, we can conclude that ART is meaningful for EC/AEH patients with fertility issues. However, there are still some controversies in terms of the safety for EC/AEH patients in making them achieve a livebirth by means of ART. Controlled ovarian stimulation (COS) during ART treatment can lead to a significant increase in estrogen level over a short period of time. Whether it will lead to an increase in the recurrence rate and selection of the best COS protocol is of concern to reproductive endocrinology and infertility (REI) doctors. In this study, we analyzed the clinical data of EC/AEH patients who received IVF to elaborate the safety of EC/AEH patients receiving ART and the factors affecting recurrence rate, in order to provide more treatment experiences for REI doctors as well as gynecologists.

## Methods

### Study Design and Patients

In this single-center retrospective study, we reviewed the medical records of infertile patients with EEC or AEH who underwent IVF after achieving CR at the Reproductive Center of Peking University Third Hospital (PUTH) between June 2010 and June 2021. Follow-up ended on October 31, 2021. This study was approved by the Ethics Committee of the PUTH (No. IRB 00006761-M2021237).

The inclusion criteria were as follows: (1) histologically proven well-differentiated endometrioid EC or AEH, magnetic resonance imaging confirmed no infiltration of myometrium; (2) accepted standard conservative therapy and achieved CR; (3) age ≤40 years old; (4) hysteroscopic evaluation performed and histologically proven normal endometrium before COS; and (5) performed standard COS protocol cycles.

**Figure 1:**
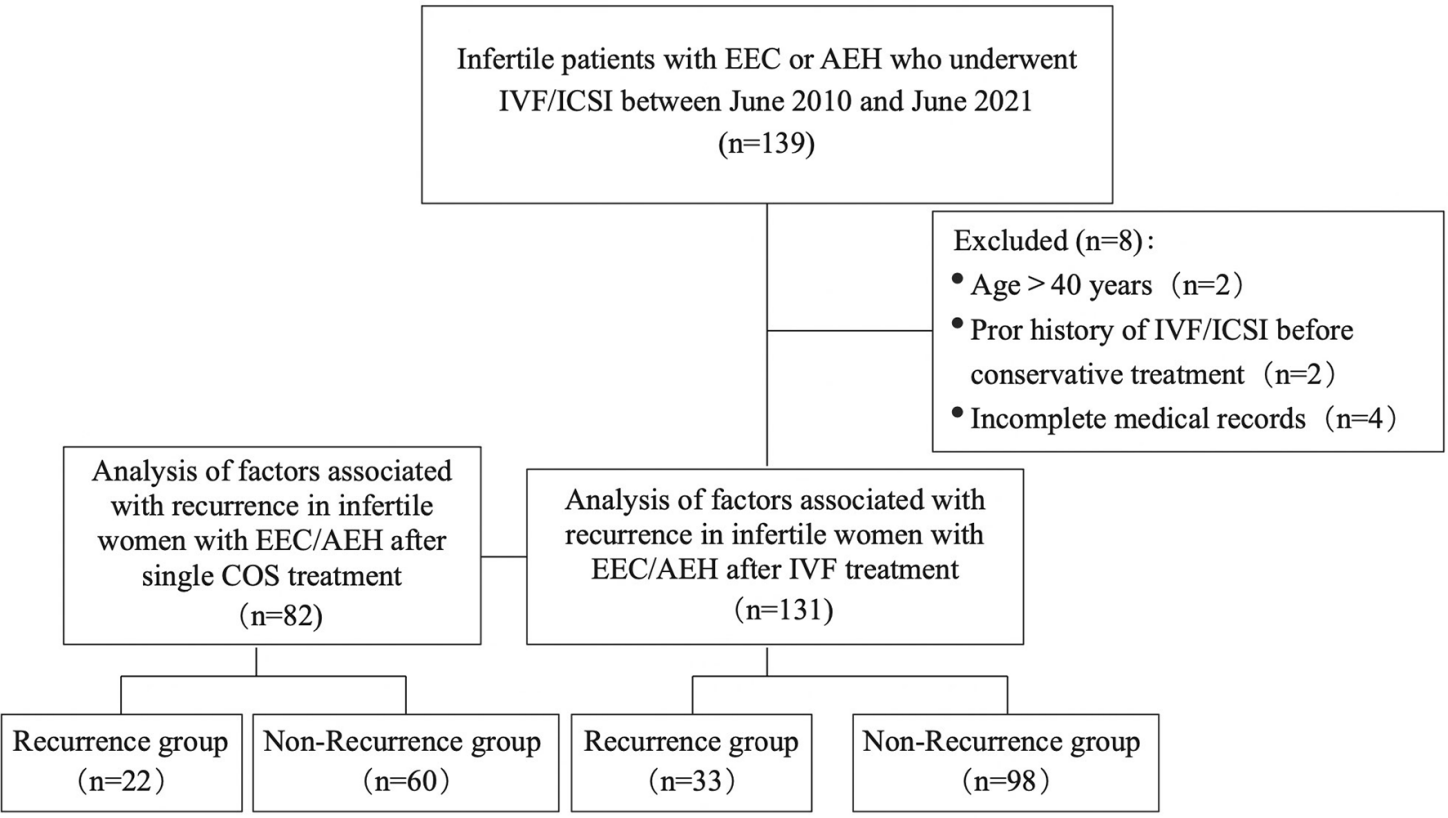
Flow chart of the analysis cohort.

The selection process of the study population is illustrated in **Figure 1**. Between June 2010 and June 2021, 139 infertile patients with EEC or AEH were referred to our reproductive center after achieving CR. Eight patients were excluded from the study for the following reasons: age >40 years old (n=2), prior history of IVF before conservative treatment (n=2), incomplete medical records (n=4). A total of 131 patients were included in the analysis. Clinical and IVF/ICSI characteristic data were reviewed and extracted from both paper and electronic medical records.

### Conservative Treatment

The endometrial lesion of each patient was comprehensively evaluated by the gynecologic oncologist and met the criteria for fertility-sparing treatment. All patients received oral progestins or intrauterine progesterone therapy including four different treatment regimens as follows: (1) MA at a dose of 160–320 mg per day (n=32); (2) MPA at a dose of 250–500 mg per day (n=95); (3) Gonadotropin-releasing hormone agonist (GnRHa)combined levonorgestrel intrauterine sustained release system (LNG-IUS) (n=2); (4) Intrauterine LNG-IUS alone (n=2). Hysteroscopy and endometrial biopsy were performed every three months to evaluate the treatment response.

Once patients achieved CR, some discontinued MA or MPA, and were referred to the REI specialists directly for ART. Some patients continued to receive the same regimen for another 3–6 months according to different doctors’ opinions, which was defined as maintenance therapy, before referral to REI specialists.

### IVF Treatment

A comprehensive evaluation of infertility was performed by a REI specialist for every patient who was referred to the reproduction center. Agonist, antagonist, or mild-stimulation protocols were used for ovarian stimulation in patients who received IVF/ICSI treatment. Gonadotropin (Gn) usage involves follicle stimulating hormone (FSH), human menopausal gonadotropin (HMG), and recombinant follitropin β injection. Agonist protocol includes three different dosage forms of GnRHa as follows: ultra-long protocol, long protocol, and short protocol.

Ultra-long protocol means intramuscular injection of 3.75 mg of long-acting GnRHa was performed on the 1st or 2nd day of the menstrual cycle, and Gn was started 30 days later until the trigger day. Long protocol means intramuscular injection of 1.25 mg long-acting GnRHa was given during the luteal phase of the previous menstrual cycle, and Gn was started 14 days later until the trigger day. Short protocol means intramuscular injection of short-acting GnRHa 0.1 mg/d was started on the 2nd day of the menstrual cycle, and Gn was started on the 3rd day of the menstrual cycle until the trigger day. Antagonist protocol means Gn was started on the 2nd day of the menstrual cycle, and 0.25 mg/d gonadotropin-releasing hormone antagonist (GnRH-A) was added when the dominant follicle diameter was 12-14 mm on the trigger day. Mild-stimulation protocol means that letrozole 2.5 or 5.0 mg/d was given orally from the 2nd to 6th day of the menstrual cycle for 5 days. Meanwhile, intramuscular injection of hMG 75-150 U/d was given from the 3rd day of the menstrual cycle. GnRH-A 0.25 mg/d was added when the diameter of the dominant follicle reached 12-14 mm, until the trigger day. The start dose of Gn is determined by the individual patient (150-300 U/d), and is adjusted according to follicular development in the process of COS.

Ovarian follicular development was monitored by TVS, and recombinant human chorionic gonadotropin (r-hCG) was administered to induce oocyte maturation when at least two leading follicles reached 18 mm in diameter. Oocyte retrieval was performed 34 and 38 h later. Oocytes were fertilized using conventional IVF or ICSI. The development and quality of embryos were assessed on day 3, as previously published, considering the percentage of fragmentation and quality of cytoplasm (10). Top-quality embryos on day 3 were defined as embryos that were derived from 2 PN embryos and could reach 5- to 8-cell stage with cytoplasmic fragmentation of <30% and even blastomeres. Non-top-quality embryos were extensively cultured to the blastocyst stage. Blastocyst morphology was evaluated on day 5 using the Gardner grading system (11). Two top-quality embryos on day 3 or one on day 5 were transferred in the fresh ET cycle. Some patients did not accept fresh ET because of the thin endometrium, a high risk of ovarian hyperstimulation syndrome, or some other reasons. Surplus viable embryos were cryopreserved according to a vitrification protocol and thawed as previously described (12). During frozen-thawed ET (FET) cycles, frozen embryos were transferred on day 3 or 5 throughout the natural or artificial cycles.

Regular luteal support was provided as oral dydrogesterone at 20 mg/d or vaginal administration of progesterone 60 mg/d from the day of ET to throughout the 10th week of gestation.

### Definition of Observation Indicators

CR was defined as the absence of hyperplasia, cancerous lesions, or other abnormal histological findings. Recurrence was defined as endometrial carcinoma or atypical hyperplasia confirmed by endometrial biopsy that recurred during ART treatment or during follow-up after ART. Treatment duration was calculated as the interval from the start to the end of oral or intrauterine progesterone treatment. The time to CR was calculated as the interval from the start of progesterone treatment to CR. The duration of maintenance therapy was calculated from the date of CR to the end of progesterone treatment. The time to IVF was defined as the interval between the CR and the start of IVF cycle. Live birth was defined as any birth event beyond 28 weeks of gestation, in which at least one neonate was born alive. The cumulative live birth rate (CLBR) of the study cohort was defined as the number of women who achieved a live birth divided by the total study population.

### Statistical Methods

Continuous data with normal distribution were represented as mean (standard deviation, SD), while continuous data with non-normal distribution were represented as median (interquartile range, IQR). Continuous data were analyzed using T test and Mann-Whitney U test. Categorical data were expressed as percentages and analyzed using Chi-square test or Fisher exact test. The median recurrence interval and cumulative recurrence rate were calculated using the Kaplan-Meier method, and the difference in recurrence rate was tested with log-rank method. COX regression model was used for correlation analysis of tumor RFS time. All analyses were performed using SPSS software (version 25.0; IBM Corp, Armonk, NY, USA). Significance was defined as a two-sided *p*-value <0.05.

## Results

### Baseline Characteristics

Up to October 31, 2021, 131 patients included in the study were followed up for 4.0-132.0 months, with a median follow-up of 50.0 months. As shown in **Table 1**, the average age of 131 patients was 33.6 ± 3.8 years and the average BMI was 26.0 ± 4.2 Kg/m^2^ with a median infertility time of 4.0 (range: 2.0-6.0) years. Most of the study participants (80.9%) were diagnosed with primary infertility, and 25 patients (19.1%) with secondary infertility. Ovulatory dysfunction and fallopian tube factors were the main causes of infertility, accounting for 38.9% (51 cases) and 24.4% (32 cases), respectively.

**Table 1:** Baseline characteristics of the analysis cohort.

Characteristics	Total (n=131)	Non-recurrence (n=98)	Recurrence (n=33)	*p* value
Age, mean (SD), years	33.6 (3.8)	33.7 (3.8)	33.6 (4.0)	0.420
BMI, mean (SD), Kg/m^2^	26.0 (4.2)	26.1 (4.1)	25.7 (4.7)	0.861
Histology type, n (%)				0.001*
EC	30 (22.9)	15 (15.3)	15 (45.5)	
AEH	101 (77.1)	83 (84.7)	18 (54.5)	
Type of infertility, n (%)				0.614
Primary	106 (80.9)	78 (79.6)	28 (84.8)	
Secondary	25 (19.1)	20 (20.4)	5 (15.2)	
Duration of infertility, median (IQR), years	4.0 (2.0-6.0)	4.0 (2.0-6.0)	4.0 (2.0-6.5)	0.924
Causes of infertility, n (%)				0.192^#^
Male factors	15 (11.5)	9 (9.2)	6 (18.2)	
Tubal factors	32 (24.4)	24 (24.5)	8 (24.2)	
Ovarian factors	51 (38.9)	41 (41.8)	10 (30.3)	
Uterine factors	5 (3.8)	2 (2.0)	3 (9.1)	
Unknown factors	28 (21.4)	22 (22.4)	6 (18.2)	
Complications, n (%)				
PCOS	33 (25.3)	30 (26.1)	9 (23.1)	0.827
DM	12 (9.2)	9 (9.2)	3 (9.1)	1.000^#^
Hypertension	7 (5.3)	5 (5.1)	2(6.1)	1.000^#^
Ovarian reserve, median (IQR)				
AMH (ng/mL)	1.1 (0.4-2.1)	1.1 (0.5-1.7)	1.2 (0.3-2.5)	0.976
No. of basal AFC	7.0 (4.0-13.0)	7.0 (4.0-14.0)	7.0 (5.0-10.0)	0.850
Basal LH, median (IQR), mIU/mL	1.8 (0.6-4.0)	1.9 (0.8-4.0)	1.7 (0.5-3.2)	0.419
Basal FSH, median (IQR), mIU/mL	6.0 (4.4-8.1)	6.1 (4.5-8.1)	6.0 (4.4-8.1)	0.824
Basal E_2_, median (IQR), pmol/L	131.0 (92.5-128.5)	128.5 (89.7-172.0)	132.0 (104.5-177.5)	0.654
No. of COS cycles, mean (SD)	1.6 (0.9)	1.6 (0.9)	1.5 (0.8)	0.521
No. of ET cycles, mean (SD)	1.8 (1.2)	1.8 (1.3)	1.9 (1.1)	0.352
Total dose of Gn, median (IQR), IU	3600.0 (2100.0-5268.8)	3550.0 (2306.3-5587.5)	3750.0 (2087.5-5025.0)	0.994
No. of days of ovarian stimulation, median (IQR)	14.0 (11.0-24.0)	14.0 (10.0-24.0)	14.0 (12.0-24.0)	0.493
With a livebirth, n (%)	66 (49.6)	57 (58.2)	9 (27.3)	0.003*

BMI, body mass index; EC, endometrial carcinoma; AEH, atypical endometrial hyperplasia; AMH, anti-mullerian hormone; AFC, antral follicle count; E_2_, estradiol; CR, complete remission; IVF, in vitro fertilization; SD, standard deviation; Gn, gonadotropin; IQR, interquartile range; FSH, follicle-stimulating hormone; LH, luteinizing hormone; PCOS, Polycystic ovary syndrome; DM, Diabetes mellitus; COS, controlled ovarian stimulation; ET, embryo transfer.

*p<0.05.

^#^Fisher’s exact test.

One hundred thirty-one patients were assigned into the recurrence (33 cases) and non-recurrence (98 cases) groups. The number of patients combined with those with polycystic ovarian syndrome (PCOS), diabetes mellitus (DM) and hypertension were 33 (25.2%), 12 (9.2%) and 7 (5.3%), respectively. However, there were no significant differences in the incidence of these complications between the recurrence group and the non-recurrence group (PCOS, DM and hypertension, *p*=0.827,1.000 and 1.000, respectively). Meanwhile, there were no significant differences in basal sex hormone levels (LH, E_2_ and FSH, *p*=0.419, 0.654 and 0.824, respectively) and basal Antral follicle count (AFC) (*p*=0.850) between the two groups.

In total, 131 patients underwent an average of 1.6 ± 0.9 COS cycles and 1.8 ± 1.2 embryo transfers (ETs), and there was no significant difference in the number of COS cycles between the recurrence group and the non-recurrence group (*p*=0.521). Each patient received 3600.0 (range: 2100.0-5268.8) IU of Gn in all COS cycles, and the total number of days of Gn injection was 14.0 (range: 11.0-24.0) days, and there was no significant difference between the two groups (*p*=0.493 and 0.352, respectively).

Recurrence occurred in 15 of 30 EC patients and 18 of 101 AEH patients, with a significantly higher recurrence rate in the EC group than in the AEH group (50.0% vs 17.8%, *p*=0.001). The proportion of patients who achieved a live birth was significantly different between the two groups (*p*=0.003).

### Characteristics of Conservative Treatment

According to **Table 2**, there were four conservative treatment regimens including 95 patients (72.5%) using MPA and 32 patients (24.4%) using MA. Both GnRHa combined with LNG-IUS and LNG-IUS alone were reported in 2 patients (1.5%), and there was a significant difference between the different regimens used in the recurrence and non-recurrence groups (*p*=0.021). The mean treatment duration was 7.2 months, and the treatment duration in the recurrence group was significantly longer than that in the non-recurrence group (8.6 vs 6.7 months, *p*=0.023). The number of hysteroscopic operations in the recurrence group was also significantly higher than that in the non-recurrence group (4.0 vs 3.0 times, *p*<0.001). The mean CR time and the median time to IVF in the recurrence group was not significantly different from that in the non-recurrence group.

**Table 2:** Characteristics of conservative treatment of the analysis cohort.

Characteristics	Total (n=131)	Non-recurrence (n=98)	Recurrence (n=33)	*p* value
Conservative treatment schedule				0.021*^#^
MPA	95 (72.5)	76 (77.6)	19 (57.6)	
MA	32 (24.4)	21 (21.4)	11 (33.3)	
GnRHa+LNG-IUS	2 (1.5)	1(1.0)	1 (3.0)	
LNG-IUS	2 (1.5)	0 (0.0)	2 (6.1)	
Treatment duration, mean (SD), months	7.2 (4.6)	6.7 (4.3)	8.6 (5.1)	0.023*
Time to CR, mean (SD), months	4.9 (2.2)	4.6 (1.8)	5.7 (3.1)	0.162
Recurrence before IVF, n (%)	19 (14.5)	11 (11.2)	8 (24.2)	0.086
Maintenance therapy before IVF, n (%)	63 (48.1)	43 (43.9)	20 (60.6)	0.110
No. of hysteroscope, median (IQR)	3.0 (2.0-4.0)	3.0 (2.0-4.0)	4.0 (3.0-5.5)	0.000*
Time to IVF, median (IQR), months	9.0 (5.0-16.0)	8.5 (4.0-16.0)	11.0 (6.0-18.0)	0.234
Clinical intervention after IVF or delivery, n (%)	18 (13.7)	14 (14.3)	4 (12.1)	1.000^#^
Time of follow-up, median (IQR), months	50.0 (31.0-80.0)	58 (37.8-86.5)	31.0 (22.5-46.0)	0.000*

EC, endometrial carcinoma; AEH, atypical endometrial hyperplasia; MPA, medroxyprogesterone acetate; MA, megestrol acetate; GnRHa, gonadotropin-releasing hormone agonist; LNG-IUS, levonorgestrel-releasing intrauterine system;IVF, in vitro fertilization; CR, complete remission; IQR, interquartile range; SD, standard deviation.

*p<0.05.

^#^Fisher’s exact test

Among 33 patients with recurrence, 12 patients with EC pathology after recurrence received comprehensive staging operation, 3 patients with AEH underwent hysterectomy, and 18 patients received conservative treatment again (12 with MPA, 3 with MA, 2 with MPA+LNG-IUS, and 1 with GnRHa+LNG-IUS). Fifteen patients achieved CR again and three were still on treatment. Three patients in the non-recurrence group underwent hysterectomy after delivery.

### Factors Associated With Recurrence

Up to October 31, 2021, 33 of 131 patients had recurrence during follow-up, with a 3-year RFS rate of 83.2 ± 3.4% and a 5-year RFS rate of 72.9 ± 4.4%. Four of 131 patients with less than 12 months of follow-up were excluded, and 127 patients were finally included in the COX regression analysis.

As shown in **Table 3**, continuous variables were converted to categorical variables based on clinical experience and related literature reports. Univariate COX regression analysis was conducted and showed that the type of histology (HR: 5.56, 95%CI: 2.73-11.33, *p*<0.001), maintenance therapy before IVF (HR: 2.03, 95%CI: 1.01-4.09, *p*=0.047) were associated with a higher recurrence rate. Patients who successfully achieved a live birth had a significantly lower recurrence rate (HR: 0.27, 95%CI: 0.12-0.58, *p*=0.001). There were no significant differences in recurrence rates among patients receiving different conservative treatments (*p*=0.080).

**Table 3:** Univariate and multiple COX regression analysis of factors associated with recurrence.

Variables	Univariate analysis		Multiple analysis	
	HR (95%CI)	*p* value	HR (95%CI)	*p* value
Age (years)		0.181		0.310
≤35	1		1	
>35	0.59 (0.28-1.28)		0.66 (0.26-1.65)	
BMI (Kg/m^2^)		0.758		0.693
≤25.0	1		1	
>25.0	1.12 (0.56-2.22)		1.06 (0.48-2.36)	
Histological type		<0.001*		<0.001*
AEH	1		1	
EC	5.56 (2.73-11.33)		4.94 (2.41-10.15)	
Basal E_2_ (pmol/L)		0.785		
≤165.0	1			
>165.0	1.11 (0.53-2.33)			
Treatment duration (months)		0.192		
≤6.0	1			
>6.0	1.60 (0.79-3.21)			
Time to CR (months)		0.382		
≤3.0	1			
>3.0	1.37 (0.68-2.75)			
Maintenance therapy before IVF		0.047*		0.209
No	1		1	
Yes	2.03 (1.01-4.09)		1.63 (0.58-4.63)	
Recurrence before IVF		0.168		
No	1			
Yes	1.75 (0.79-3.88)			
Time to IVF (months)		0.637		0.530
≤3.0	1		1	
3.0-6.0	1.02 (0.28-3.81)		0.74 (0.16-3.41)	
6.0-9.0	1.74 (0.47-6.48)		1.70 (0.39-7.35)	
>9.0	1.63 (0.56-4.81)		0.85 (0.24-3.06)	
Conservative treatment		0.080		
MPA	1			
MA	1.92 (0.91-4.05)			
GnRHa+LNG-IUS	7.50 (1.71-32.86)			
LNG-IUS	2.15 (0.29-16.11)			
Total dose of Gn (IU)		0.711		
≤3600.0	1			
>3600.0	1.14 (0.57-2.26)			
Total days of ovarian stimulation		0.586		0.969
≤14.0	1		1	
>14.0	1.21 (0.61-2.39)		2.03 (0.55-7.49)	
No. of COS cycles		0.521		0.368
≤1	1		1	
>1	0.79 (0.38-1.63)		0.32 (0.08-1.23)	
With livebirth		0.001*		0.003*
No	1		1	
Yes	0.27 (0.12-0.57)		0.30 (0.14-0.65)	
Clinical intervention after IVF or delivery		0.582		0.646
No	1		1	
Yes	0.75 (0.26-2.13)		1.30 (0.38-4.45)	

BMI, body mass index; EC, endometrial carcinoma; AEH, atypical endometrial hyperplasia; E_2_, estradiol; AMH, anti-mullerian hormone; CR, complete remission; IVF, in vitro fertilization; Gn, gonadotropin; COS, controlled ovarian stimulation; IVF, in-vitro fertilization; CI, confidence interval; HR, hazard ratio; MPA, medroxyprogesterone acetate; MA, megestrol acetate; GnRHa, gonadotropin-releasing hormone agonist; LNG-IUS, levonorgestrel-releasing intrauterine system.

*p<0.05.

The number of COS cycles, basal E_2_ level, total dose of Gn, and total days of ovarian stimulation had no significant effect on the recurrence rate of EC/AEH (*p*=0.521, 0.785, 0.711, and 0.586, respectively).

**Figure 2:**
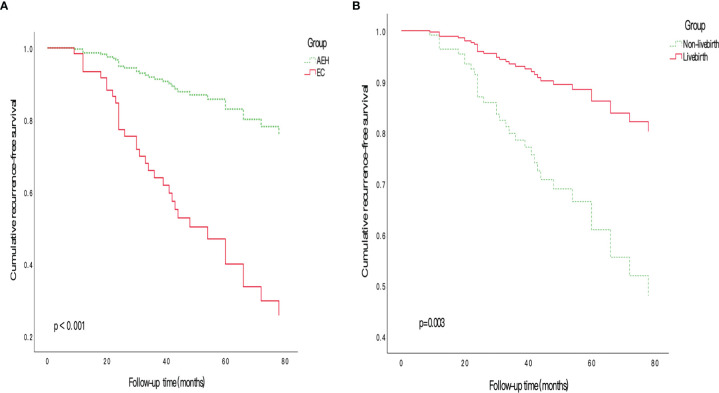
Cumulative RFS curves in fertility-sparing EEC/AEH patients after ART **(A)** The cumulative RFS in patients of AEH group and EC group. Patients had longer RFS with histology of AEH than patients with EC. **(B)** The cumulative RFS in patients of livebirth group and non-livebirth group. The cumulative RFS in patients who got a child successfully was longer than patients failed to get a child. AEH, atypical endometrial hyperplasia; ART, assisted reproductive technology; EEC, early stage endometrial cancer; RFS, recurrence-free survival.

As shown in **Table 3**, we included 9 variables (age, BMI, histology type, number of COS cycles, maintenance treatment before IVF, total days of ovarian stimulation, time to IVF, with livebirth, and clinical intervention after IVF and delivery) based on the COX univariate regression analysis, clinical experience, and published literature into COX regression model for multivariate analysis, and found that histology type (HR: 4.94, 95%CI: 2.41-10.15, *p*<0.001) and livebirth or not (HR: 0.30, 95%CI: 0.14-0.65, *p*=0.003) were independent influencing factors of recurrence. The influence on RFS of EC or AEH, for livebirth or not are shown in **Figure 2**.

### Different COS Protocols and Tumor Recurrence

As shown in **Figure 1**, 82 of 131 patients received a single COS cycle, they included 64 (78.0%) AEH patients and 18 (22.0%) EC patients. These 82 patients were summarized and analyzed using different COS protocols.

82 patients were followed up for 13.0-128.0 months. By the time of follow-up, 22 of the 82 patients had recurrence, and the 3-year RFS rate was 81.0 ± 4.6%, and the 5-year RFS rate was 73.6 ± 5.5%. As shown in **Table 4**, all 82 patients received conventional COS protocols, among which more patients adopted agonist protocol and antagonist protocol, accounting for 47.6% and 40.2%, respectively. Different protocols had no significant effect on recurrence (p=0.683). The start dose and total dose of Gn in the recurrence group were slightly higher than those in the non-recurrence group; however, the difference was not significant (212.5 vs 200.0 IU, *p*=0.797; 2650.0 vs 2550.0 IU, *p*=0.802). In addition, there was no significant difference in serum E_2_ level on the trigger day between the two groups (3880.5 vs 4678.5 pmol/L, *p*=0.530).

**Table 4:** Protocols of COS and data of IVF of 82 EC/AEH patients treated with single COS cycle.

Characteristics	Total (n=82)	Non-recurrence (n=60)	Recurrence (n=22)	*p* value
Protocols of COS, n (%)				0.683
GnRH agonist	39 (47.6)	27 (45.0)	12 (54.5)	
GnRH antagonist	33 (40.2)	26 (43.3)	7 (31.8)	
Mild stimulation	10 (12.2)	7 (11.7)	3 (13.6)	
Starting dose of Gn, median (IQR), IU	200.0 (150.0-300.0)	200.0 (150.0-300.0)	212.5 (150.0-300.0)	0.797
Total dose of Gn, median (IQR), IU	2587.5 (1751.9-3618.8)	2550.0 (1725.0-3600.0)	2650.0 (1856.3-3706.3)	0.802
Total days of ovarian stimulation, median (IQR)	11.0 (10.0-13.0)	11.0 (9.3-13.0)	12.0 (10.8-13.0)	0.194
E_2_ on trigger day, median (IQR), pmol/L	4572.0 (2369.8-8789.0)	4678.5 (2406.0-8927.0)	3880.5 (2051.0-7498.0)	0.530
No. of retrieved oocytes, median (IQR)	10.0 (4.0-14.0)	10.0 (4.3-13.8)	10.0 (3.0-15.0)	0.937
Fertilization, n (%)				0.181^#^
IVF	57 (69.5)	39 (65.0)	18 (81.8)	
ICSI	25 (30.5)	21 (35.0)	4 (18.2)	
Rate of good-quality embryos per cycle, mean(SD), %	74.7 (28.0)	73.3 (28.3)	78.7 (27.5)	0.405
No. of ETs, median (IQR)	1.0 (1.0-2.0)	1.0 (1.0-2.0)	1.0 (1.0-2.0)	0.131

COS,controlled ovarian stimulation; E_2_, estradiol; IVF, in vitro fertilization; GnRH, gonadotropin-releasing hormone; IQR, interquartile range; ICSI, intracytoplasmic sperm injection; SD, standard deviation; ET, embryo transfer.

^#^Fisher’s exact test.

As shown in **Table 5**, we included 8 variables (age, BMI, histology type, protocols of COS, total dosage of Gn, E_2_ level on trigger day, maintenance treatment before IVF, and with livebirth or not) into the COX regression model for multivariate analysis, and found that histology type (HR: 4.48, 95%CI: 1.74-11.57, *p*=0.002) and with live birth or not (HR: 0.33, 95%CI: 0.12-0.87, *p*=0.024) were independent influencing factors of recurrence. Different protocols had no significant effect on the recurrence of EC/AEH (*p*=0.326).

**Table 5:** Analysis of factors associated with recurrence for patients treated with single COS cycle.

Variables	Univariate analysis		Multiple analysis	
	HR (95%CI)	*P* value	HR (95%CI)	*p* value
Age (years)		0.995		0.942
≤35	1		1	
>35	1.00 (0.42-2.40)		1.09 (0.36-3.28)	
BMI (Kg/m^2^)		0.699		0.889
≤25.0	1		1	
>25.0	1.18 (0.51-2.73)		1.87 (0.57-6.12)	
Histological type		<0.001*		0.002*
AEH	1		1	
EC	6.08 (2.37-15.61)		4.48 (1.74-11.57)	
Treatment duration (months)		0.248		
≤6.0	1			
>6.0	2.27 (0.97-5.34)			
Time to CR (months)		0.990		
≤3.0	1			
>3.0	1.01 (0.43-2.33)			
Maintenance therapy before IVF		0.004*		0.059
No	1		1	
Yes	3.79 (1.54-9.35)		2.17 (0.47-10.17)	
Recurrence before IVF		0.463		
No	1			
Yes	2.51(1.07-5.88)			
Time to IVF (months)		0.139		
≤3.0	1			
3.0-6.0	0.57 (0.10-3.43)			
6.0-9.0	1.37 (0.23-8.19)			
>9.0	2.28 (0.66-7.90)			
Protocols of COS, n(%)		0.738		0.326
GnRH agonist	1		1	
GnRH antagonist	0.73 (0.28-1.85)		0.31 (0.08-1.20)	
Mild stimulation	1.15 (0.32-4.08)		0.26 (0.04-1.70)	
Starting dose of Gn (IU)		0.924		
≤200.0	1			
>200.0	0.96(0.42-2.22)			
Total dose of Gn (IU)		0.986		0.889
≤2500.0	1		1	
>2500.0	0.99 (0.43-2.30)		0.89 (0.23-3.47)	
Total days of ovarian stimulation (days)		0.991		
≤12	1			
>12	1.00(0.41-2.45)			
E_2_ on trigger day (pmol/L)		0.425		0.468
≤4500.0	1		1	
>4500.0	0.71 (0.31-1.65)		0.89 (0.23-3.47)	
With livebirth		0.004*		0.024*
No	1		1	
Yes	0.25 (0.10-0.65)		0.33 (0.12-0.87)	
Clinical intervention after IVF or delivery		0.634		
No	1			
Yes	0.74 (0.22-2.52)			

BMI, body mass index; EC, endometrial carcinoma; AEH, atypical endometrial hyperplasia; E_2_, estradiol; CR, complete remission; IVF, in vitro fertilization; Gn, gonadotropin; COS, controlled ovarian stimulation; IVF, in-vitro fertilization; CI, confidence interval; HR, hazard ratio.

*p<0.05.

### Pregnancy Outcomes

In total, 66 of the 131 patients achieved a livebirth, with a CLBR of 50.4% (66/131). Fifty-six cases achieved livebirths by IVF/ICSI method and 6 cases through natural pregnancy after ART termination, as well as 3 by preimplantation genetic diagnostic (PGD) cycle, and 1 by *in vitro* maturation (IVM) method. Five delivered twins and four delivered twice, giving birth to 75 live babies.

## Discussion

In this single-center retrospective study, we shared our experience of IVF treatment in patients with EEC or AEH after conservative treatment. To the best of our knowledge, this is one of the largest studies to focus on IVF treatment and recurrence outcomes of patients with EEC or AEH.

According to current reports, the overall recurrence rate of EC/AEH after conservative treatment is 35.0-62.2% (13, 14), and the recurrence rate of EC/AEH patients after ART treatment is 21.0-47.0% with median recurrence time of 12-28 months (15–19). In this study, the recurrence rate was 25.2% and the median recurrence time was 31.0 (range: 22.5-46.0) months, which is consistent with current reports on recurrence in EC/AEH patients after ART treatment. Also, the overall recurrence rate is not significantly higher than that of EC/AEH patients who received conservative treatment. This once again confirmed the safety and necessity of ART for EC/AEH patients. Also, this study reaffirmed that endometrial cancer is an independent risk factor for recurrence, which is consistent with studies have been reported. In general, the higher the grade of the lesion, the longer the patient needs to receive conservative treatment, and the more frequent intrauterine operations are required to evaluate the endometrial lesion during this period. So it explains why our analysis found that the conservative regimen duration and the number of hysteroscopic operations were higher in the recurrence group.

Although many studies have confirmed the safety of ART (20, 21), there are still many opposing opinions that COS may increase the recurrence of EC/AEH lesions (22, 23). It is well known that the COS process involves the use of high dosage Gn, and the level of serum estrogen is supraphysiological which may lead to the recurrence of the tumor lesion. Therefore, there is no definite conclusion on the choice of COS protocols for EC/AEH patients. Most REI doctors tend to choose COS protocols which combine letrozole with Gn and can reduce the estrogen level during COS process for EC/AEH patients with reproductive needs (24). However, the mild stimulation protocol usually has lower oocyte retrieval rate and fewer available embryos, and the possibility of a satisfactory pregnancy outcome is relatively low (25). Kalogiannidis proposed that GnRH-a can be used for conservative treatment of AEH due to its inhibitory effect on the endometrium, and long-term down-regulation can reduce the large dose drug accumulation effect on progeny (26). Considering this opinion, GnRH-a protocol may be beneficial in preventing the recurrence of EC/AEH lesions. However, Ichinose reported that the high level of serum estrogen after ovulation induction in EC/AEH patients did not increase recurrence (17). In our study, 82 patients who received only one COS cycle were screened for correlation analysis between different COS protocols and recurrence and it was found that compared to the mild stimulation protocol recommended by most scholars, there was no significant difference in recurrence rate among the three protocols (*p*=0.326). It can be considered that in terms of COS protocols for EC/AEH patients with reproductive needs, REI doctors have more choices based on oocyte retrieval rate, available embryos rate, clinical pregnancy rate, and live birth rate.

Our study found that the recurrence rate for patients with multiple COS cycles was not higher than that for patients with single COS cycles. We can consider that increase in COS cycles will not lead to an increase in the recurrence rate of tumor lesions. Current studies suggest that the recurrence of endometrial lesions requires long-term stimulation of estrogen, while estrogen levels only show short-term increases during COS, thus not increasing the risk of recurrence (17, 27), which supports the conclusion of our study. Of course, the analysis of COS frequency in this paper still has some limitations. Tumor outcomes were not analyzed for specific different times, but were only done with the classification of single and multiple times due to the limitation of sample. Currently, the number of EC/AEH patients receiving ART in a single center is relatively small. In future, multi-center clinical studies should be carried out to expand the sample size and provide more reliable evidence for current research theories.

Gn plays an important role in the process of COS, which is a key step in ART, to promote the development of dominant follicles and increase the number of oocytes retrieved. No matter the difference in frequency of COS or different COS protocols, it can be reflected in the duration and dose of Gn treatment in the ART process. It has been proven that different usage of Gn will lead to a great difference in serum E2 level, which may affect the outcomes of IVF pregnancy as well as the recurrence of tumor lesion (28). But there was no research analyzed the correlation of the use of Gn and recurrence of tumors in EC/AEH patients. Our analysis showed that there was no significant correlation between the total dose of Gn, duration of Gn used, basal E_2_ level on trigger day, and tumor recurrence. This result supports that the routine COS protocols in EC/AEH patients with reproductive needs does not increase the risk of tumor recurrence.

Many studies have reported the relationship between pregnancy and tumor recurrence, and found that the recurrence rate in patients who achieved live births was significantly lower than that in patients who did not, suggesting that pregnancy is a protective factor for tumor lesions (16, 21, 29). Similarly, among the 131 patients in this study, the recurrence rate of patients without live births is significantly higher than that of patients with live births (36.9% vs 13.6%, p=0.003). The result was consistent with literature reports. On one hand, the high level of progesterone during pregnancy as well as delivery and the complete decidual detachment from the uterus in the puerperium played a role similar to shaving the tumor lesions, which may prevent the recurrence of the lesion. On the other hand, pregnant women with obesity and PCOS can avoid exposure to estrogen alone for a certain period of time and delay the tumor recurrence and progression (29, 30). However, the above theory is our speculation. There was no study that confirmed that the mechanism of pregnancy can prevent the recurrence of EC/AEH lesions.

Patients were followed up by a gynecologic oncologist after childbearing. We suggest the patient undertake standard management including hysterectomy and bilateral salpinogo-oophorectomy. However, if the patients still have a strong desire to preserve their fertility, they can choose to regularly take short-acting contraceptives or intrauterine LNG-IUS. And these patients are supposed to be followed up every three months. In our study, only two patients in the non-recurrence group underwent surgery after childbirth. Therefore, we consider that most patients are unwilling to undergo the hysterectomy. Due to the limited sample size, although we found that there was no significant effect of clinical intervention after IVF or delivery on tumor recurrence, the optimal management of these patients remains to be explored.

This study had some limitations. First, this was a retrospective study conducted in a single center; therefore, selection bias may have occurred. Second, conservative treatment of EC is mostly limited to patients with lesions confined to the endometrium. It is unknown whether the moderately-differentiated tumor, invasion of the muscle layer, and tumor size will affect the prognosis of EC patients after IVF treatments. Prospective studies with larger sample sizes are needed to answer these questions in the future.

## Conclusion

The degree of the progression of an endometrial lesion into carcinoma is a key factor affecting recurrence in EC/AEH patients after IVF/ICSI treatment; successful live birth is a protective factor against the recurrence of endometrial lesions. Different COS protocols and COS frequencies, as well as different doses and duration of Gn used during ART, did not affect the recurrence of endometrial lesions.

## Data Availability Statement

The raw data supporting the conclusions of this article will be made available by the authors, without undue reservation.

## Ethics Statement

The studies involving human participants were reviewed and approved by Ethics Committee of the Peking University Third Hospital (No: IRB 00006761-M2021237). Written informed consent for participation was not required for this study in accordance with the national legislation and the institutional requirements.

## Author Contributions

YG, XZ, HL, and JQ developed the design of the study. YG and XZ collected the clinical data. YG drafted the manuscript and contributed the data analysis. XZ, HL, and JQ proofread and revised the manuscript. All authors read and approved the final manuscript.

## Funding

This study was supported by grants from CAMS Innovation Fund for Medical Sciences(2019-I2M-5-001) and the National Key Research and Development Program of China (2018YFC1002101).

## Conflict of Interest

The authors declare that the research was conducted in the absence of any commercial or financial relationships that could be construed as a potential conflict of interest.

## Publisher’s Note

All claims expressed in this article are solely those of the authors and do not necessarily represent those of their affiliated organizations, or those of the publisher, the editors and the reviewers. Any product that may be evaluated in this article, or claim that may be made by its manufacturer, is not guaranteed or endorsed by the publisher.
